# Trabecular deformations during screw pull-out: a micro-CT study of lapine bone

**DOI:** 10.1007/s10237-017-0891-9

**Published:** 2017-03-06

**Authors:** Thomas Joffre, Per Isaksson, Philip Procter, Cecilia Persson

**Affiliations:** 1 Materials in Medicine Group, Division of Applied Materials Science, Department of Engineering Sciences, Uppsala University, Uppsala, Sweden; 2 Applied Mechanics, Department of Engineering Sciences, Uppsala University, Uppsala, Sweden

**Keywords:** Screws, Implants, Trabecular bone, Pull-out test, Digital volume correlation, Micro-CT

## Abstract

The mechanical fixation of endosseous implants, such as screws, in trabecular bone is challenging because of the complex porous microstructure. Development of new screw designs to improve fracture fixation, especially in high-porosity osteoporotic bone, requires a profound understanding of how the structural system implant/trabeculae interacts when it is subjected to mechanical load. In this study, pull-out tests of screw implants were performed. Screws were first inserted into the trabecular bone of rabbit femurs and then pulled out from the bone inside a computational tomography scanner. The tests were interrupted at certain load steps to acquire 3D images. The images were then analysed with a digital volume correlation technique to estimate deformation and strain fields inside the bone during the tests. The results indicate that the highest shear strains are concentrated between the inner and outer thread diameter, whereas compressive strains are found at larger distances from the screw. Tensile strains were somewhat smaller. Strain concentrations and the location of trabecular failures provide experimental information that could be used in the development of new screw designs and/or to validate numerical simulations.

**Electronic supplementary material:**

The online version of this article (doi:10.1007/s10237-017-0891-9) contains supplementary material, which is available to authorized users.

## Introduction

Screws are one of the most commonly used orthopaedic implants worldwide. They are used both for the fixation of complicated bone fractures as well as for fixation of other implants. However, in 2–40% of patients these screws migrate and/or loosen with failure of the surrounding bone as the main reason (Cornell 2003; Norris et al. 2012). Failure rates are increasing, and this has been linked to the ageing population leading to a higher number of patients presenting osteoporosis-induced low bone quality (Riggs and Melton 1995). Typically screws are inserted either into cortical bone or into cancellous bone where there is an overlying cortical shell. Reduced cortical shell thickness in the metaphysis of elderly osteoporotic patients is usually also associated with lower bone mineral density (BMD) in the underlying cancellous bone (Rausch 2013). In cancellous bone, there is very little bone in contact with the screw. Typically apparent densities are higher than 10% for healthy bone and lower than 5% for poor bone quality. Therefore, when a hole is drilled into such a structure, it results in an inner surface with a very limited capacity for transferring loads. It has been suggested that when the overlying cortical shell is thicker than 1.5 mm, it accounts for most of the holding power of a screw (Seebeck 2005).

Cements have been used together with screws as a means to improve load transfer (Procter 2015; Larsson 2012; Choma 2012; Fölsch 2012; Stadelmann 2010; Juvonen 2015; Kainz 2014), but they do not always improve the pull-out strength, as illustrated by, e.g. the study conducted by Procter (2015), where the augmented screws gave a lower pull-out force than non-augmented screws in 4 out of 21 human femoral bone specimens. This work suggests that there are other factors affecting the pull-out strength, such as positioning of the screw with respect to the bone morphology and that the pull-out force is highly sensitive to initial conditions such as the exact positioning, the insertion depth or the angle of insertion. Chapman (1996) studied screw pull-out in vitro in both foam model materials as well as in calf and human vertebral bone. They suggested that thread “shape factor”, i.e. the arithmetic product of pitch and thread depth, is an important factor and that decreased thread pitch increases screw purchase strength in a porous material. A 3D finite element (FE) study by Kamane (2016) examined the influence of different screw parameters on predicted screw pull-out and concluded that smaller thread pitch was linked to higher screw pull-out forces, further emphasising the importance of the screw design. Another study by Gausepohl (2001) compared cancellous, cortical and “machine” (fine thread and pitch) screws in synthetic and bovine bone. They conclude that some features of natural bone cannot be adequately simulated in homogeneous artificial test materials. Additionally that fine machine screws had an advantageous relation between thread diameter and purchase and that could be due to local micro-impaction of bone fragments. They caution that predrilling of the screw holes should be avoided in cancellous bone as it weakens the screw/bone interface. On the issue of local compaction, a so-called spring back effect was observed by Kold (2003): in an in vivo canine study, they showed that compaction leads to dimensional changes in drilled holes. They conclude that a reduction in implant-to-bone gaps is associated with increased fixation strength. The thread geometry, self-tapping (or not) or alternatively use of a drill and the drill to minor diameter logic, the degree to which any of these contribute to compaction are all variables that make rigorous understanding of the fixation of screw implants challenging in trabecular bone. The heterogeneous and complex microstructure leads to complex mechanical interactions between the screw and the bone, and previous studies on this topic have shown that models considering the trabecular bone as a continuum fail to predict the strain field and more generally the mechanical behaviour of a screw implant (Wirth et al. 2012a).

The bone anisotropy and microstructural variability between patients complicate the design of screw implants: different screw designs may give different results in different subjects since each piece of trabecular bone is unique. Yet, novel screw designs are commonly evaluated according to ASTM F 543 (Conshohocken 2013) using a rigid polyurethane foam with a regular structure as specified ASTM F 1839 (Conshohocken 2001). Thus, the optimal design of a cancellous screw for a given microstructure remains an open problem. A computational model may provide a cost and time-efficient way to evaluate and optimise novel screw designs in a range of different bone microstructures.

Several numerical models have been developed over the last few years aiming to predict and understand the deformation that occurs during a pull-out test of a screw implant. However, due to the complex microstructure, different simplifying assumptions have been adopted, such as the use of a cylinder as simplified screw geometry (Ruffoni et al. 2012), a regular grid to simplify the bone microstructure (Brown 2013), and perfect bonding between the screw and the bone (Wirth et al. 2012a, b; Rungsiyakull 2015). Moreover, the screw insertion operation itself is likely to induce micro-fractures in the bone structure that may substantially lower the bone’s mechanical performance (Yadav 2012; Wang 2014), something that has not been taken into consideration in the aforementioned studies.

All the computational models mentioned above lack a direct experimental validation that could confirm whether or not the simplifying hypotheses are valid. Indeed, even though the finite element method is a well-known and established method for modelling structural problems, the presence of a heterogeneous anisotropic microstructure together with a bone/metal interface and complicated contact load distributions makes it a challenging problem. Experimental validations are necessary to further development and ensure validity of the simulation results. Only one study, by Wirth (2010), compared the results of a numerical model with mechanical pull-out tests and was able to validate the strength predictions of the model but not the stiffness predictions, indicating a need for further improvement in this area.

The aim of this study was to increase the understanding of induced frictional effects and local deformation fields in the bone structure occurring during a screw pull-out test. To this end, pull-out tests were carried out in the chamber of an X-ray microtomographic scanner. The experimental set-up allowed acquisition of sequential images of the deformation that occurs in trabeculae due to the displacement of the screw during the pull-out test. An image correlation method, digital volume correlation (DVC), was then used to estimate the strain field inside the bone and thus quantify the observed mechanisms. DVC has already been proven useful in quantifying the deformation in trabecular bone alone (Gillard 2014; Roberts et al. 2014). However, in previous studies on the topic (Gillard 2014), the voxel size of the images was greater than $24\, {\upmu }\hbox {m}$ and, as a result, the deformation was measured rather at a mesoscale and not at the lower trabecular level. In this study, a voxel size of $6.5\, {\upmu }\hbox {m}$ was used, which allowed for illustration and quantification of the motion of the screw and the bone, as well as an estimation of the type of deformation occurring in the trabeculae.

**Fig. 1:**
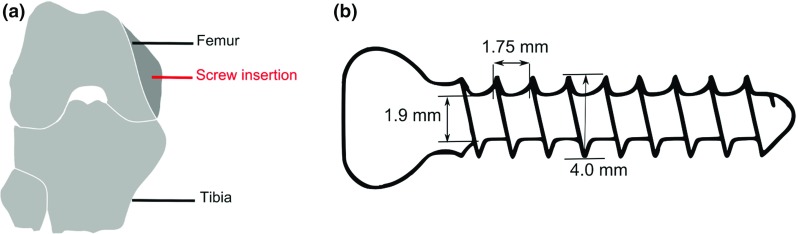
Schematics of **a** screw insertion into rabbit femur, **b** key dimensions of a screw

**Fig. 2:**
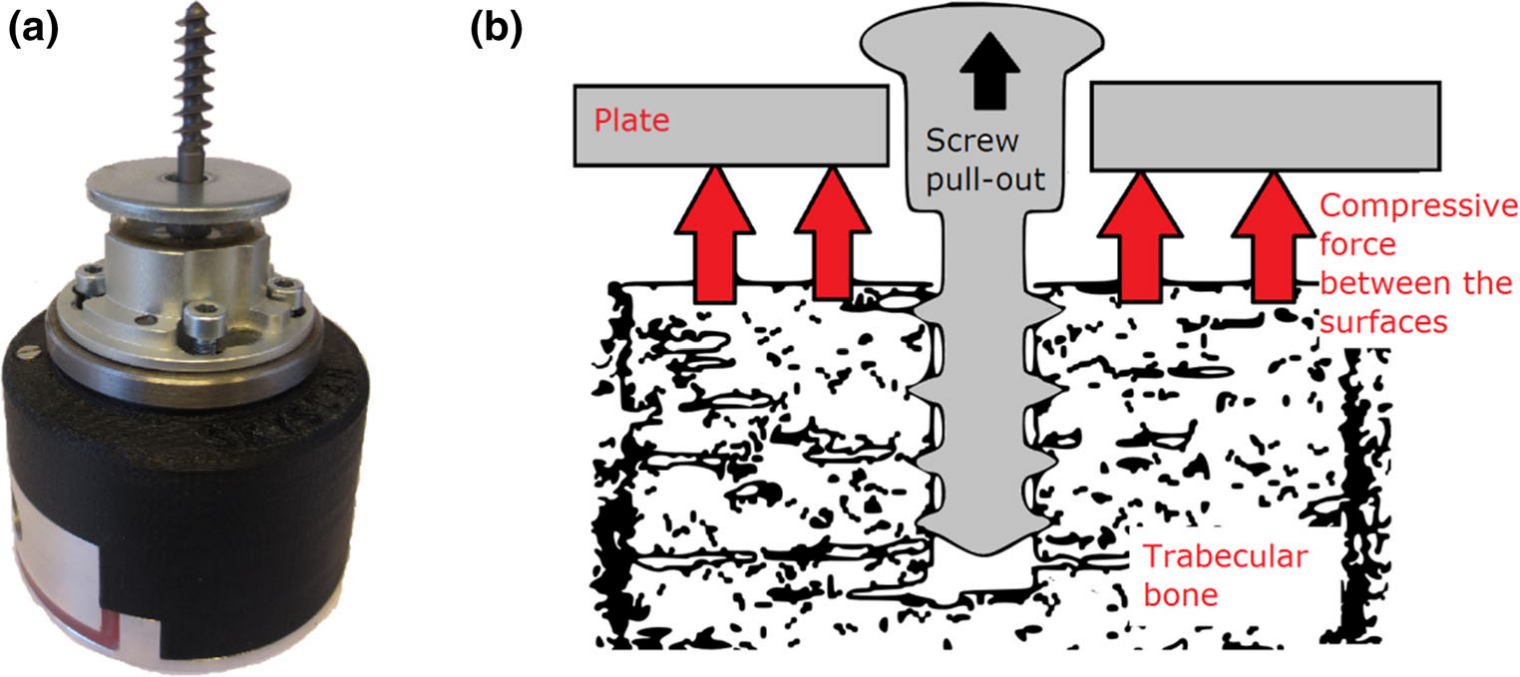
**a** Loading device adapted for in situ mechanical testing. The screw in the picture has a diameter of 6 mm. **b** Scheme showing the principle of the pull-out experiment

## Materials and methods

### Sample preparation

Commercial bone screws (Jiangsu Trauhui Medical Instrument Co., China) were implanted into lapine femoral bone (Fig. 1). The screws had an outer diameter of 4.0 mm (HB 4.0, ASTM F 543–07). The key dimensions of the screws are indicated in Fig. 1b.

The cortical shell of the femur was first removed using a bone saw. Then machine held drilling was conducted into the trabecular bone, using a drill of a diameter of 2 mm, which represents overdrilling the minor diameter of the screw by 0.1 mm (Fig. 1b). The screws were then inserted into the trabecular bone to a depth of 9 mm.

### Mechanical testing

In situ X-ray computational tomography experiments were performed on four samples (from four different animals), to investigate the mechanical behaviour of the trabecular bone during screw pull-outs. The equipment used, a SkyScan-1172, has a resolution high enough to distinguish individual pores and trabeculae. The apparatus has a built-in tensile stage that was used to pull-out the inserted screws. The load cell used had a capacity of 440 N. During the tests, the samples were mounted on a holder with a possibility of rotation, allowing for X-rays to enter from different directions. In this way, two-dimensional projection images were taken in a multitude of directions, which allowed subsequent reconstruction of 3D microstructures. During each scan, 900 radiographs were taken over 360$^{\circ }$ with a pixel size of $6.5 \,{\upmu }\hbox {m}$. The screws were pulled out stepwise allowing for successive 3D images showing the complete deformation process in the bone structure.

**Fig. 3:**
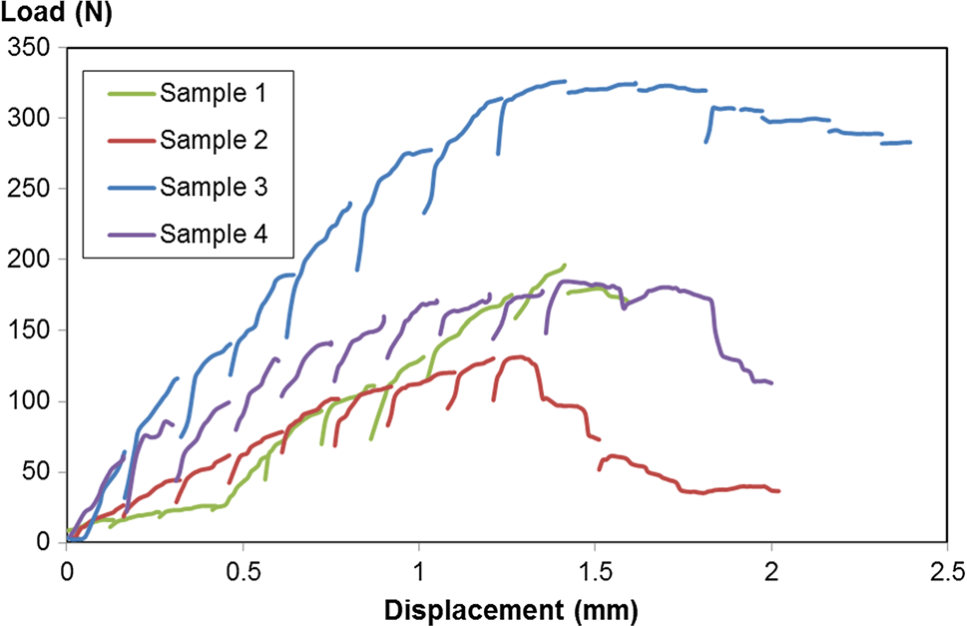
Load versus displacement during the test. The tests were interrupted for about 90 min for image acquisitions at every 0.15 mm. Note that the discontinuities in the curves are due to relaxation during these interruptions

The standard method of pull-out experiments, as described in ASTM F543 (Conshohocken 2013), could not be directly applied, because of (1) the small size of the chamber in the ${\upmu }\hbox {CT}$, (2) no metallic objects can be in the path of the X-ray during the scan, and (3) the motor of the loading stage could not reach the standard speed of 5 mm/min. A metal plate with a hole (with a diameter twice as large as the outer diameter of the screw) was used to prevent motion of the bone when the screw was pulled out (see Fig. 2). The displacement speed was 0.3 mm/min during loading. At every load increment of 0.15 mm, the tests were interrupted for about 90 min for image acquisitions. Some relaxation phenomena occurred during these interruptions, leading to a small decrease in image quality and discontinuities in the force–displacement curves (see Fig. 3).

### Digital volume correlation

The images acquired during the in situ experiments were cross-correlated using a digital volume correlation technique (Bay 1999). The volume was digitally divided into sub-volumes, each of them characterised by a number of geometrical features making it unique when compared to the other sub-volumes in the same test specimen. For most materials, a random or regular pattern, which deforms along with the object, must be applied to the specimen, when performing these types of image analyses. However, for trabecular bone there is no need for this since the inherent variations in density and thickness of the trabeculae are a sufficient reference pattern (see e.g. Gillard 2014; Dall’Ara et al. 2014). However, there is a lower limit in terms of the size of each sub-volume: the sub-volume must include enough features to make it distinguishable so that tracking is possible. Here, a size of 32 voxel per sub-volume was chosen.

The method used is briefly discussed here, and a more thorough description can be found in, e.g. Forsberg (2010) and Forsberg (2008). It should be noted that the method described here is a local method. For the interested reader, we refer to the study by Palanca (2015), where a comparison between the local and global approach based on elastic registration is made. A Lagrangian formulation is applied to describe the mechanical behaviour of the structural components. Let the position vector $${\varvec{X}}$$ refer to a position of a point in the body in its initial (reference) configuration, and let the position vector $${\varvec{x}}$$ refer to the position of the same point in the body in its deformed configuration. Both $${\varvec{X}}$$ and $${\varvec{x}}$$ are described in a 3D Cartesian (*X*, *Y*, *Z*) coordinate system. Now, to be able to estimate the three-dimensional deformation field from the acquired images, the following strategy is applied: denote the initial and deformed configuration of a sub-volume as $$S_1 $$ and $$S_2$$, respectively. Without any deformation, the sub-volume $$S_2$$ would be an exact copy of the sub-volume $$S_1 $$, if the noise during image acquisition can be neglected. A mechanical load applied to the body may result in deformation of the sub-volume, described by the vector $${\varvec{u}}$$, i.e.

1 $$\begin{aligned} {\varvec{u}}={\varvec{x}}-{\varvec{X}}. \end{aligned}$$

A convenient way to estimate the displacements between the reference and deformed body (the sub-volume $$S_1 $$ and $$S_2 )$$ using two acquired images is to minimise a three-dimensional discrete function $$\varOmega $$ using all sub-volumes (Forsberg 2010), according to:

2 $$\begin{aligned} \varOmega =1-\frac{\int {S_1 ({\varvec{X}}+{\varvec{u}})S_2 ({\varvec{X}})\hbox {d}V} }{\left[ {\int {S_1^2 ({\varvec{X}}+{\varvec{u}})S_2^2 ({\varvec{X}})\hbox {d}V} } \right] ^{1/2}}, \end{aligned}$$

where *V* is the volume of the scanned body. Chebyshev polynomials of the first kind are chosen as basis to describe the estimated deformation field ${\varvec{u}}$ in the body, cf. Forsberg (2008). The algorithm for computing (2) was implemented in a MATLAB code (Forsberg 2010). The Green–Lagrange strain *E* is readily obtained using (1) and

3 $$\begin{aligned} {\varvec{E}}=\frac{1}{2}[{\varvec{F}}^{T}{\varvec{F}}-{\varvec{I}}], \end{aligned}$$

where ${\varvec{I}}$ is the unit matrix, and ${\varvec{F}}=\partial {\varvec{x}}/\partial {\varvec{X}}$ is the deformation gradient. The deformation gradients here have an explicit solution since the deformation field ${\varvec{u}}$ is decomposed using Chebyshev polynomials of the first kind (Forsberg 2008). The three principal strain components $E_{\mathrm{I}} \ge E_{\mathrm{II}} \ge E_{\mathrm{III}}$ are the eigenvalues of the strain ${\varvec{E}}$, i.e. the roots $\lambda$ to the equation

4 $$\begin{aligned} |{\varvec{E}}-\lambda {\varvec{I}}\,\,| =0. \end{aligned}$$

The third principal strain $E_{\mathrm{III}}$ hence corresponds to the highest estimated compressive principal strain in the material. The first principal shear strain $\gamma _{\mathrm{I}}$ is estimated via the relationship $\gamma _{\mathrm{I}} =[E_{\mathrm{I}} -E_{\mathrm{III}} ]/2$. Finally, some methods to improve accuracy were adopted. An overlap of 50% between the sub-volumes was used: the correlation windows (sub-volumes) overlap each other by 16 voxels (half the side of the correlation window) in each direction. Thus, each voxel in the full correlated region, except those near the borders, is a member of eight individual correlation windows. The final displacement field in a given sub-volume is then calculated as a weighted sum according to

5 $$\begin{aligned} {\varvec{u}}(x,y,z)=\frac{1}{m}\sum _{i=1}^m {{\varvec{u}}_i (x_i ,y_i ,z_i )/r_i^2 } . \end{aligned}$$

**Table 1:** Maximum load and displacement at maximum load for all tested specimens

	Maximum load (N)	Displacement at max. load (mm)	Bone volume fraction (calculated using boneJ, Doube 2010)
Sample 1	186	1.26	0.26
Sample 2	134	1.25	0.22
Sample 3	314	1.35	0.30
Sample 4	184	1.41	0.29
Average (±SD)	205 (${\pm }66$)	1.32 (${\pm }0.07$)	0.27 (${\pm }0.03$)

The insertion depth was $9 \pm 0.2\,\hbox {mm}$

where $r_i$ is the distance to the *i*th sub-volume centre normalised so that $\sum {r_i^{-2} =1}$ and *m* is the number of overlapping sub-volumes. An alternative to using eqn. (5) would be to take smaller steps between the sub-volumes and only pick the central value from each of those. However, in our study this alternative strategy resulted in too small volumes for the correlation analysis to provide good values (i.e. the volumes did not have enough distinguishable features). For a thorough description of the methodology, please consult Sjödahl and Oreb (2002). Since the screws are much stiffer than the trabecular bone, the strains in the screws were assumed to be negligible.

### Precision test

To estimate the error in strain map determined using DVC, the procedure initially proposed by Liu and Morgan (2007), and later adapted and completed by Dall’Ara et al. (2014), was used: after a first original scan, a second one was taken in the same initial position. The second scan was then virtually moved by 2 voxels in each direction (hence simulating a rigid body motion). Dall’Ara et al. (2014) have shown that a rigid body registration has a very limited effect compared to the noise due to acquisition, emphasising the importance of performing the accuracy test on two different scans. The displacement and strain between the two scans were then estimated using the method described in the previous section. This experiment was then used to estimate the noise in strain values: since no loads are applied, the strain tensor should be zero everywhere. The error in strain was then considered to be the average of the absolute value of the six components of the strain tensor.

### Image registration

Due to the difference in stiffness between the titanium screw and the trabecular bone, it can be assumed that the screw is not deforming during the pull-out test. However, when pulling out the screw, the bone might rotate and unscrew itself since rotation of the bone is not constrained and some (frictional) movement is likely to occur at the bone–screw interface. To quantify this motion, the screw was registered on the images such that it occupied the exact same position at the different loading steps. The procedure consisted of three steps. First, specific points along the screw were identified and matched against each other. Secondly, any rigid body motion was determined which minimised the square of the pairwise distances between the points. Then, rigid body transformation of the mechanical system was made using standard transformation rules.

## Results and discussion

### Loads and displacements during pull-out

**Fig. 4:**
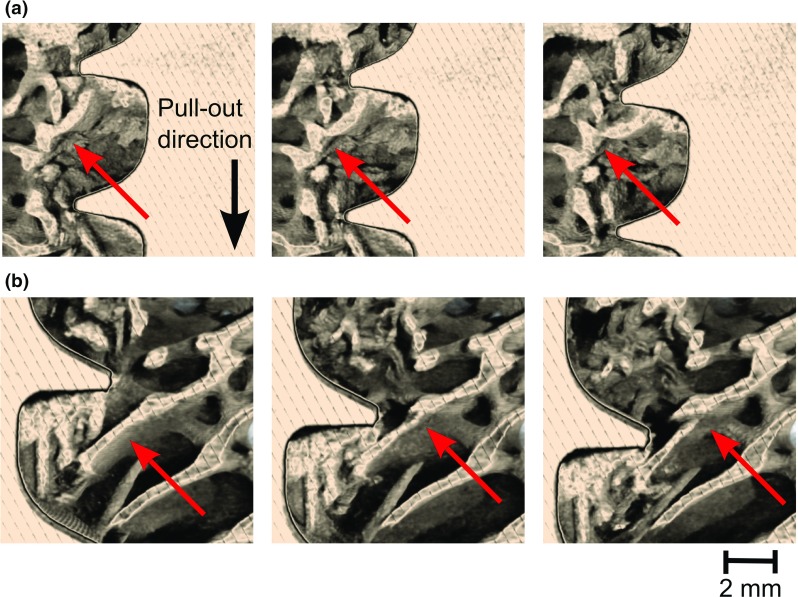
Sequential images of the failure of individual trabeculae **a** the trabecula is bending and failing at approximately 0.8 mm from the thread **b** the trabecula is failing close to the screw thread. The dimensions of the screw are given in Fig. 1. The *red arrows* are pointing towards the point where the failure occurred

The results highlighted the high variability in strength and stiffness of different bone–implant constructs (Table 1; Fig. 3). The measured maximum load ranged from 134 to 314 N. Figure 3 also indicates that some material relaxation occurred during the scanning, which is a limitation of the present method: the relaxation is likely to reduce the measured forces, and the estimated force values are, hence, not directly comparable with other studies where the standard ASTM F543 has been followed. However, despite the apparent relaxation the acquired images were not blurry (c.f. Fig. 4) and no loss of image quality could be observed during the pull-out (see videos provided in supplementary information), which suggests that no significant motion occurred within the scanning time at the length scale of the detector pixel size ($6.5 \,{\upmu }\hbox {m}$). For these reasons, the relaxation is here assumed to have a negligible effect on the displacement and strain estimations. In a previous study on rabbit femur (Larsson 2012), the reported values for the pull-out strength were above 500 N, using the same screw, but the cortical shell was not removed. This highlights the importance of the cortical shell in the fixation, in agreement with Thiele (2007). However, when studying the fixation of the screw in trabecular bone, removing the cortical shell avoids the effects due to the variation of density and thickness of the cortical layer. The bone volume fraction varied between the samples (Table 1), which is likely to influence the measured pull-out forces. However, this variation alone cannot account for all the variations between the samples, and there was no statistically significant ($p=0.42$, $R^{2}=0.61$) correlation between the pull-out strength and the bone volume fraction.

### Screw and bone motion

Three videos are provided in the supplementary information to this article showing sequential images of the pull-out of the screw from the bone. In the first two videos, the motion is observed in the coordinate system of the $\hbox {X}{\upmu }\hbox {CT}$. In the third video, the screw position is fixed using the registration method described in Sect. 2.4. Firstly, these videos give an overview of how far from the screw the trabeculae are actually deforming and failing. In Fig. 4, two different failures of trabeculae are illustrated. In Fig. 4a, the trabecula is breaking at approximately 1 mm from the screw thread, whereas in Fig. 4b, the trabecula is breaking at the screw/bone interface. The separation of trabecular failure into these two categories could be important to improve the design of cancellous screws and to compare screws with similar diameters but different threads: the failures similar to Fig. 4a could be less related to the thread design since the bone is breaking far away from the thread. The failures such as the one illustrated in Fig. 4b are related to the direct contact between a trabecula and the thread and thus could be influenced by thread design parameters such as the angle of the thread. To optimise the mechanical performance of a screw thread, the number of trabeculae failing at the interface thread/bone probably has to be reduced: if the loads are better transmitted to the bone microstructure, strain concentrations and thus potentially stress concentrations will be less important at the screw/bone interface, limiting the number of trabeculae likely to fail due to the high shear strains below the thread. However, more information about the failure mechanisms of single trabeculae is needed to validate this assumption. In this particular study, 30 single trabecula failures were randomly selected from the 4 different samples and sequential images similar to Fig. 4 were extracted. A manual measurement (performed with the software ImageJ) of the distance between the thread and the approximated location of the bone failure revealed that both failure mechanisms are significant: 18 of the failures were located less than 0.5 mm from the screw surface and were attributed to a direct contact with the screw, whereas 12 were located deeper inside the bone microstructure and were related to a bending of the trabeculae due to a load applied by the thread on another part of the trabeculae.

**Fig. 5:**
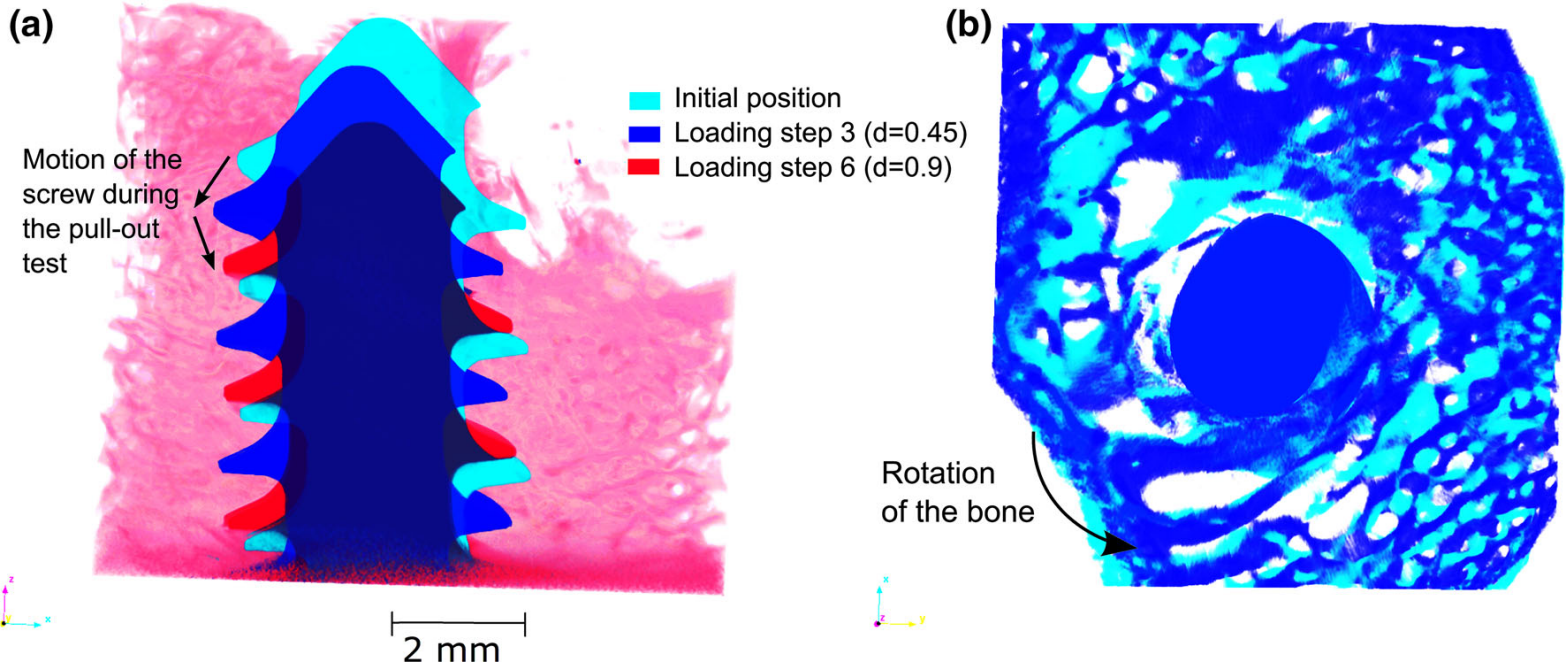
Motion during the pull-out test: **a** motion of the screw (in the $\hbox {X}{\upmu }\hbox {CT}$ coordinate system) **b** top view of the motion of the bone with respect to the screw position (registered images), illustrating the unscrewing of the bone during the pull-out experiment

Figure 4 also illustrates the difficulties in modelling this problem. Due to the screw insertion, the trabeculae are damaged, and some pieces of broken bone are present which may affect the mechanical performance as pointed out by Gausepohl (2001) and Steiner et al. (2016). This damage is outwith the capability of a FE model to simulate. Studies where the screw is virtually inserted on an $\hbox {X}{\upmu }\hbox {CT}$ image of the undrilled trabecular bone are thus likely to overestimate the contact area between the screw and the bone at a given displacement. It can also be observed that the screw is not retracting in a straight line during the pull-out test (Fig. 5a). This is not due to a misalignment of the screw during the insertion, since the screw is not moving in a different direction at every displacement increment. The density variation within the sample and the damage induced during the drilling result in a variation in contact and stiffness of the bone throughout the length of the thread. As a result, the screw tends to slightly move from one to another direction. This result illustrates the absence of symmetry of the problem: to model the pull-out experiments, the whole length of the screw and the 3D nature of the microstructure of the bone should be taken into consideration: the heterogeneities of the microstructure are affecting the screw motion. This phenomenon might be accentuated here and thus more visible due to the overdrilling of 4% performed before the screw insertion. Previous studies on this topic have usually been conducted using $\hbox {Sawbones}^{\mathrm{TM}}$ (Ramaswamy et al. 2010; Yánez et al. 2010; Patel et al. 2010), a rigid polyurethane foam with a regular structure (as specified in ASTM F 1839), which is likely to reduce this phenomenon, partly invalidating its use as test material to assess the performance of a specific screw design.

Another key observation is the rotation of the bone that occurs during the test (Fig. 5). From the registration of the screw position on the images, it appears that the bone is rotating approximately 8$^{\circ }$ (${\pm }3^{\circ }$) during the pull-out test. This confirms the fact that friction plays an important role in the pull-out mechanisms. It also supports the result obtained numerically for bone anchors by Hughes (2014): a low friction between the thread and the bone can lead to a slight rotation of the bone anchor during pull-out. The use of bone cement or an adhesive might suppress this rotation and lead to a stiffer response of the implant.

**Fig. 6:**
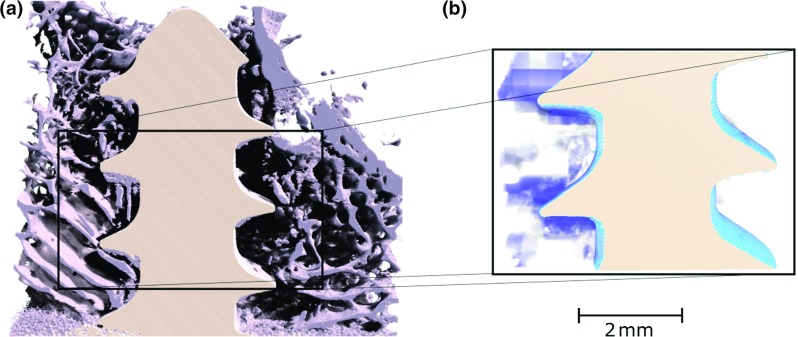
**a** 3D visualisation of half of the screw during the pull-out test and **b** zone with the third compressive strain $(E_{\mathrm{III}})$, evaluated with DVC, inferior to −0.05

### Strain measurements

**Fig. 7:**
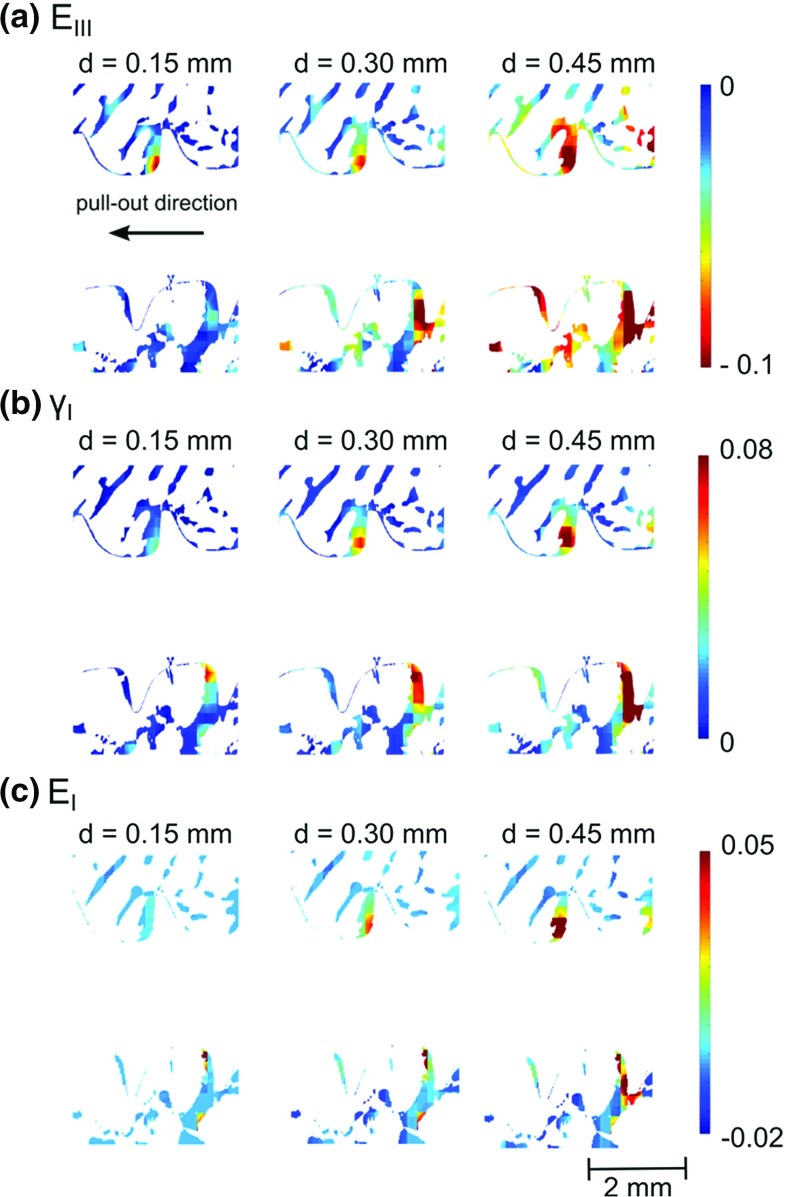
2D visualisation (based on the 3D calculations) of **a** the compression zone around the screw $({E}_{\mathrm{III}})$, **b** first principal shear strain $({\varvec{\gamma }}_\mathbf{I} )$, and **c** first principal strain $({E}_{\mathrm{I}})$ at different displacements. All the results are plotted in the initial configuration

The average strain estimated during the precision test was $325\times 10^{-6} \,({\pm }184\times 10^{-6})$, which is slightly lower than the one reported by Liu and Morgan (2007) (who reported values from $380\times 10^{-6})$ but higher than Gillard (2014) reported less than $200\times 10^{-6}$. To obtain a higher precision, a larger sub-volume would have to be used. Even though the estimated values are in the same range as that generally reported in the literature, the displacement of the screw implant introduces high strain levels at the vicinity of the thread, both because of the contact loads themselves but also due to frictional effects along the thread surface. The error related to the presence of high strain gradients is not assessed by the precision test, which is based on a pure rigid body motion. Thus, the actual error close to the thread is likely to be higher than the values indicated above. Therefore, the strains estimated close to the thread (i.e. at a distance inferior to approximately 0.5 mm) are given mainly for qualitative analysis since their precision is hard to evaluate. The sub-volume used in this study is only slightly smaller than the average thickness of the rabbit trabeculae (208 vs $220 \,{\upmu }\hbox {m}$, Cao 2004). Therefore, the strains are estimated at the trabecular level but due to this size limitation, the method does not permit a clear visualisation of strain variations within the trabecular thickness. The discussion is thus focused on the type of deformation and on the volume of bone undergoing significant deformation. However, the results could still be used to validate FE models by, e.g. comparing the displacement field of the centre of the sub-volume with the displacement field obtained in a numerical model (as pointed out by Chen 2016). In Fig. 6a, a 3D visualisation of the whole screw before failure is plotted. The region showing strains $E_{\mathrm{III}} <-0.05$ are presented in Fig. 6b. It can be noticed that the end of the screw is close to the cortical layer due to the rather small size of the rabbit femur, which is also the case in a previous study (Larsson 2012). From the 3D visualisation of strain (Fig. 6), the area of interest, i.e. the zones where high deformation occurs, was identified, and the bone trabecular structure was used as a mask to plot the deformation in 2D (see Fig. 7). Even though here $E_{\mathrm{III}}$ is plotted as an example, it should be underlined that the full strain tensor is estimated with the DVC, and the information provided includes also information about the direction of principal strain or high shear zones.

**Fig. 8:**
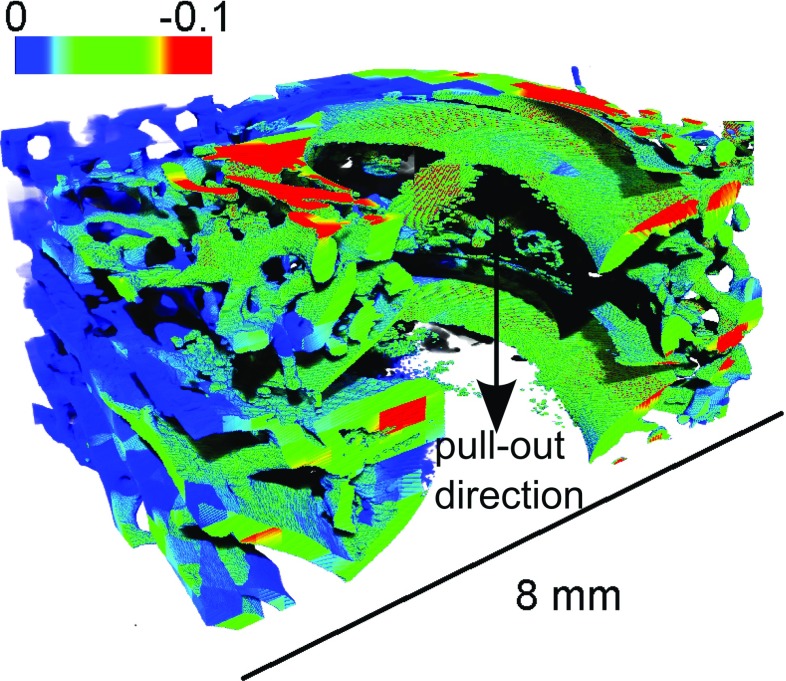
3D visualisation of compression zone around the screw $({E}_{\mathrm{III}})$ for the sample 4, the displacement of the screw was 0.45 mm. The screw has been removed virtually to improve the visualisation

**Fig. 9:**
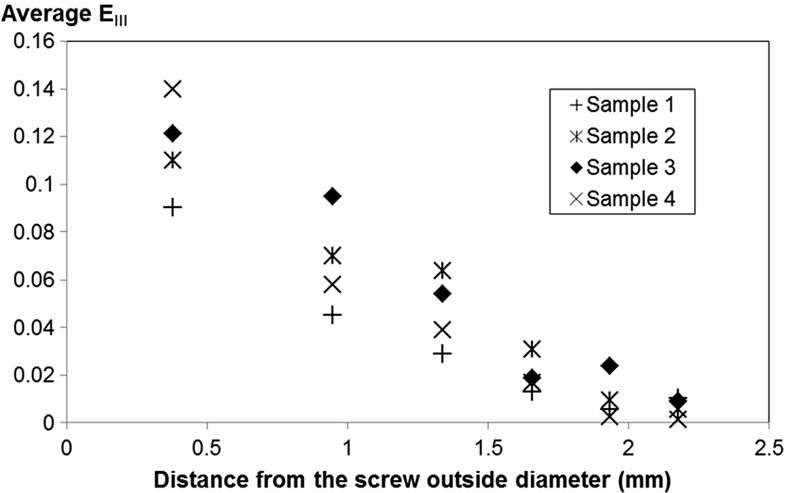
Average third principal strain $({\varvec{E}}_{\mathbf{III}})$ versus distance to the centre of the screw at a displacement of 0.6 mm

As expected, the deformation in the trabecular bone was initiated just below the thread and then increased radially inside the bone, in a direction out from the screw, as the screw was pulled out. Some bending of the trabeculae could be quantified (see Fig. 7c, trabeculae in the bottom right) confirming the observation made in Fig. 4. The resulting strain field was much more heterogeneous (see Fig. 7) than the one usually obtained through FE simulation (see e.g. Wirth et al. 2012a; Wirth 2010): the strain here is much more confined and concentrated in the vicinity of the thread. Another important result given by the DVC is how far deformations are present radially in the trabecular bone (see Fig. 9). A previous study (Wirth et al. 2012a), using a cancellous screw of the same outer diameter inserted in various human bone, has shown numerically that the strain in the bone is still significant (approximately 10% of the maximum strain) at a distance of approximately 6 mm from the screw. This suggests that in a simulation, the bone has to be modelled with the correct microstructure at least up to a distance of 6 mm from the outer diameter of the screw. Here the experimental results suggest a much faster decrease in the compressive strains (Figs. 8, 9). This might be due to the assumption of perfect bonding (no friction) between the screw and the bone used in simulations, which might lead to an overestimation of the number of load-carrying trabeculae. It should also be mentioned as a source of discrepancy that rabbit bone is used in the current study, which might affect these results due to the differences in density, trabecular thickness and spacing compared to human bone (Doube et al. 2011). The predrilling diameter might also influence this result since it affects the pull-out force (Chapman 1996). The strain concentration generated by the thread could also be used to compare different threads: a different thread design, giving a better distribution of the loads, might lead to lower strain concentrations for the same applied displacement. Here, an example is sample 3, which gave a much stiffer response than the other samples (see Table 1). First, this might be due to the higher estimated bone volume fraction, but additionally it can be observed in Fig. 9 that compressive strains at a distance of 1 mm from the screw outer diameter are much higher in this sample than in the other samples. This might indicate a better initial positioning of the screw leading to a better distribution of the loads in the microstructure of the bone and could also explain the high pull-out force measured on this sample.

When comparing the highest shear strain to the compressive strains, it appears that the shear strains are much more concentrated just inside the major diameter, whereas compressive strains are present deeper inside the microstructure. The high shear strain might be the result of the angle of the screw thread and friction at the screw/bone interface, something that could be taken into account in the design of novel screws. For instance, Kamane (2016) concluded that a smaller thread pitch lead to higher pull-out forces. But by changing the pitch, they also modified the angle of the thread, and thus both the pitch and the angle of the thread might have improved the stability of the implant.

Another important point illustrated in the videos, Figs. 4 and 7, is that the trabeculae are entering in contact with the bone at different displacements. Thus, an accurate simulation of a pull-out test should also take into consideration that not all the trabecular bone enters into contact with the screw at the same displacement; this would become even more important in less dense cancellous structures than those seen in healthy bone.

## Conclusions

The current study enabled 3D visualisation of the interaction between trabeculae and screw threads during pull-out testing of a screw. This enabled a three-dimensional insight into how trabeculae are irreversibly deforming and failing during pull-out of the screw. It was observed that approximately 60% of trabeculae failed at the screw/bone interface, whereas 40% failed deeper inside the bone that is adjacent to the screw. Digital volume correlation gives us some insight about the type of deformation occurring in areas having a size close to the trabecula thickness. The results showed that the highest shear strains are confined to just below the screw thread, whereas compressive strains extend deeper inside the microstructure, up to a distance of approximately 2 mm (half the screw diameter). The high shear strain below the thread appeared to be related not only to friction effects but also to the inclination of the screw thread, which could be taken into account during the development of new screw designs aiming to reduce strain concentrations between the inner and outer thread. The results may also be used for validation purposes of numerical simulations.

## Electronic supplementary material

Below is the link to the electronic supplementary material.

Supplementary material 1 (mp4 5768 KB)

Supplementary material 2 (mp4 4559 KB)

Supplementary material 3 (mp4 3778 KB)
